# Rare presentation of maxillary osteonecrosis and tooth exfoliation induced by herpes zoster infection in a 29-year-old Chinese male: a case report and literature review

**DOI:** 10.1186/s12903-024-04641-8

**Published:** 2024-07-27

**Authors:** Jian Sun, ZhenPeng Zhao, Jianfeng He

**Affiliations:** 1 Department of Stomatology, The First Affiliated Hospital, Zhejiang University School of Medicine, Hangzhou, 310003 Zhejiang Province People’s Republic of China; 2 The People’s Hospital of Zhaoyuan City, Zhaoyuan, 265400 Shandong Province People’s Republic of China

**Keywords:** Herpes zoster, Osteonecrosis, Tooth exfoliation, Varicella-zoster virus, Trigeminal nerve

## Abstract

**Background:**

We present a case of a 29-year-old male patient without immunodeficiency who suffered from rapid osteonecrosis and tooth exfoliation resulting from herpes zoster (HZ) infection in the left maxillary branch of the trigeminal nerve. Various complications associated with shingles infections have been reported, cases of osteonecrosis and tooth exfoliation due to HZ infection among young people without immunodeficiency are rare. In this case, we focus on the particular manifestation of HZ infection.

**Case presentation:**

The patient presented with clusters of erythema and papules, along with non-hemorrhagic blisters on the left face and the loss of the left upper incisor. All lesions were localized to the left side of the face without exceeding the midline. After receiving antibacterial and antiviral treatment, successful control over the infection was achieved; however, he experienced the loss of all upper teeth on the left side except for the first and second upper left molars.

**Conclusion:**

This case highlights that rapid osteonecrosis and tooth exfoliation may occur among young individuals without immunodeficiency after HZ infection. HZ infection of the face should be taken very seriously to obtain prompt treatment to prevent the rare complications of bone necrosis and tooth loss as much as possible.

## Background

Herpes zoster (HZ) infection, commonly known as shingles, is a viral disease that occurs with varicella-zoster virus (VZV) reactivation. The symptoms typically start with pain along the affected dermatome, followed by a vesicular eruption in 2–3 days [1]. The disease has an incidence rate of 1 in 1,000 among young individuals and exhibits a 5-10-fold increase in the elderly population [2]. The onset of HZ can be triggered by various factors, including trauma, malignant lesions in the dorsal root ganglion, exposure to X-rays, or immunosuppressive therapy, and predisposing factors include HIV infection [3, 4], certain types of malignancies accompanied by compromised immune responses, notably Hodgkin’s disease, lymphatic leukemia [5], and kidney transplant recipients [6]. The most commonly affected sites are the thoracic dermatomes (T3-L3, 56%) and the trigeminal ganglia (∼ 20%); among the three branches of the trigeminal nerve, it is most frequently observed that the ophthalmic nerve branch is impacted [7]. While HZ can cause complications like postherpetic neuralgia, it is not typically associated with osteonecrosis or tooth exfoliation. Once the maxillary or mandibular are compromised, complications such as alveolar bone necrosis or tooth exfoliation may arise [8–11]. In the present article, we describe a case of HZ infection in a 29-year-old male patient without underlying systemic conditions who presented with rapid maxillary tooth exfoliation followed by subsequent osteonecrosis.

## Case presentation

**Fig. 1:**
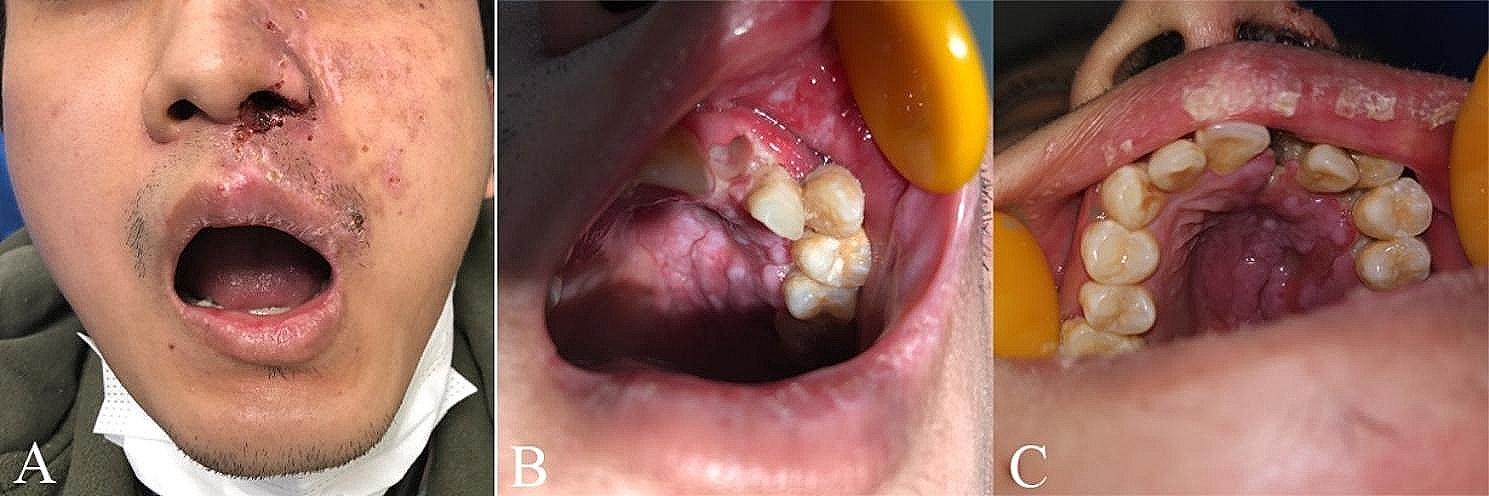
(**A**) Extraoral photograph (frontal view) showed multiple irregular, crusted lesions on the left face extending along the rami maxillary trigeminal nerve. (**B**) Intraoral photograph of the empty extraction fossa with smooth inherent alveolar bone, devoid of bleeding, and several large ulcerative surfaces in the mucosa. (**C**) Intraoral photograph captured swelling, congestion, and erosion in the upper left hard palate

On March 24, 2021, a 29-year-old male patient visited our outpatient clinic with a complaint of recent loss of the left central upper incisor within the past twelve hours. According to the patient’s medical history, clusters of erythema and papules, along with non-hemorrhagic blisters, emerged from a small blister on the left side of the face and upper palate, corresponding to the distribution area of the maxillary branch of the trigeminal nerve 15 days before the teeth loss. The papules and erythema displayed a linear distribution pattern. A burning sensation was experienced in both the left side of the face and interior of the upper palate, progressing into painful ulcerations. Although severe swelling was not observed in the upper left side of the face and orbital area, pigmentation and crusts were present on the left side of the nose, upper lip, and local cheeks (Fig. 1A). Laboratory tests revealed a neutrophil count of 9.47 × 10^9^/L, with a reference range of 2-7 × 10^9^/L, and a monocyte count of 1.18 × 10^9^/L, with a reference range of 0.2-1.0 × 10^9^/L. The total protein level was 96.4 g/L, the reference range was 65–85 g/L, and the globulin level was 50.7 g/L with a reference range of 20 to 40 g/L.

**Fig. 2:**
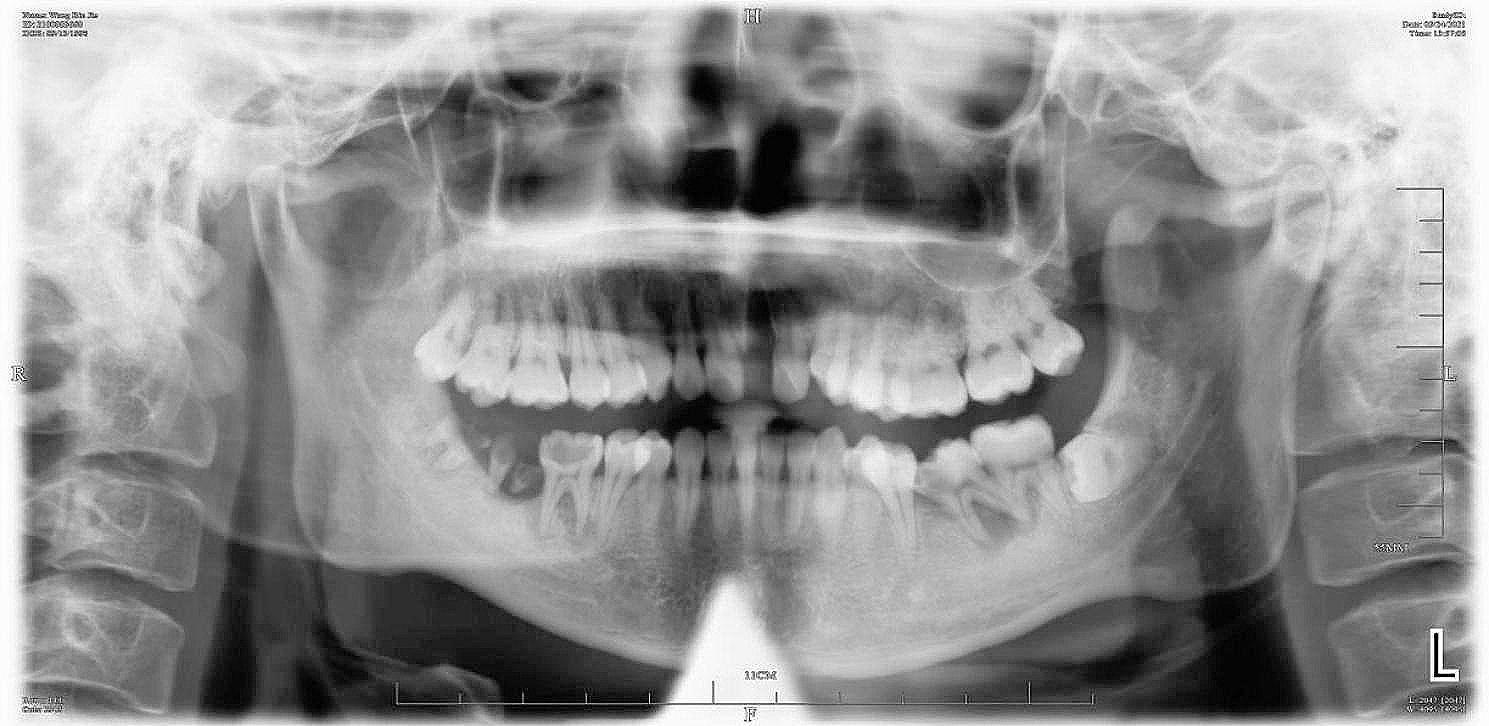
Panoramic radiographs on the first visit

The patient had no previous history of bisphosphonic acid therapy, radiotherapy, or any significant systemic diseases. However, he experienced chickenpox due to a lack of vaccination during childhood. Extraoral examination revealed multiple irregular superficial sores and scabs on the left lip and perioral skin, including the upper lip, red lip, nostrils, nasal wing, and suborbital region. The left nostril exhibited scabbing and exudation (Fig. 1A). Intraoral examination revealed the presence of multiple diffuse ulcers of varying sizes on the labial mucosa and mucosa of the upper lip on the side (Fig. 1B). Severe erythema, edema, and congestion were observed in the upper left gum, along with evident periodontal pyorrhea. The upper left hard palate displayed folds indicative of swelling, congestion, and erosion (Fig. 1C). The left upper incisor was absent, exposing a smooth alveolar bone in the socket without any signs of bleeding or purulent exudates. Radiological examination revealed an alveolar cavity in the upper left jaw with no evidence of necrotic bone (Fig. 2). Importantly, it should be noted that the lesion did not extend beyond the midline in either intraoral or extraoral areas.

The preliminary diagnosis was made based on the patient’s medical history and clinical data, indicating HZ affecting the left side of the face with involvement of the maxillary branches of the trigeminal nerve. The patient was prescribed famciclovir (0.25 g administered three times daily for 7 days), mecobalamin (0.5 mg administered three times daily for 24 days), gabapentin (300 mg taken twice daily for 10 days), cefuroxime (250 mg taken twice daily for 7 days), and metronidazole (0.2 g administered three times daily for 10 days).

**Fig. 3:**
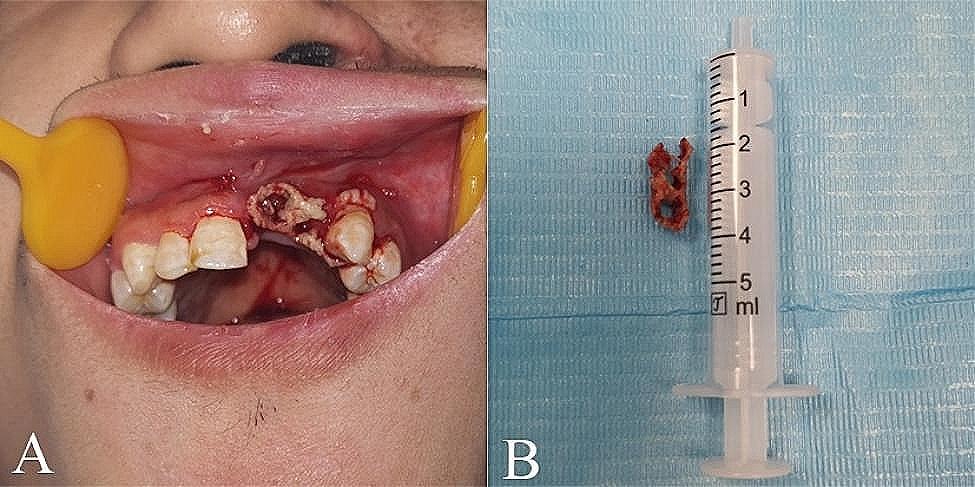
(**A**) The intraoral photograph showed the presence of sequestrum formation in the area of the left upper anterior teeth. (**B**) The removed sequestrum

**Fig. 4:**
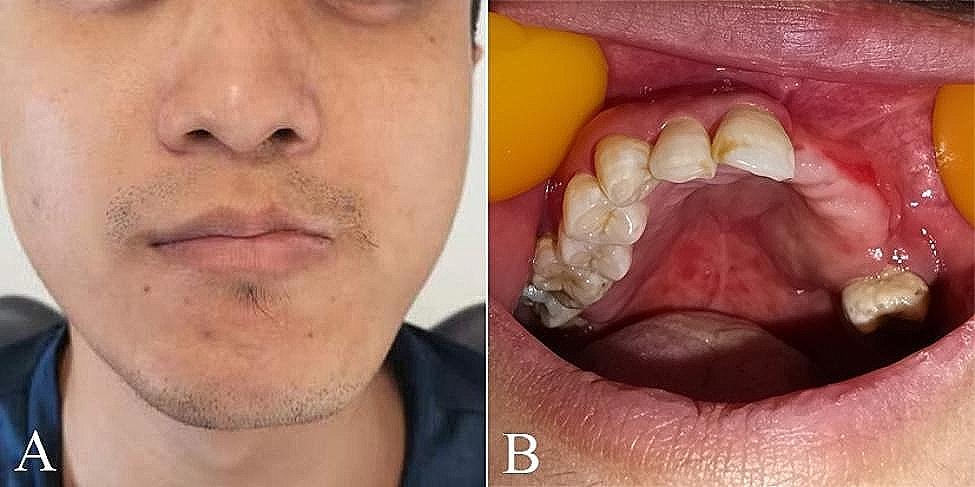
(**A**) The extraoral photograph showed the facial scars and hyperpigmentation mostly subsided. (**B**) The intraoral photograph showed the full recovery of mucous membranes and gums

**Fig. 5:**
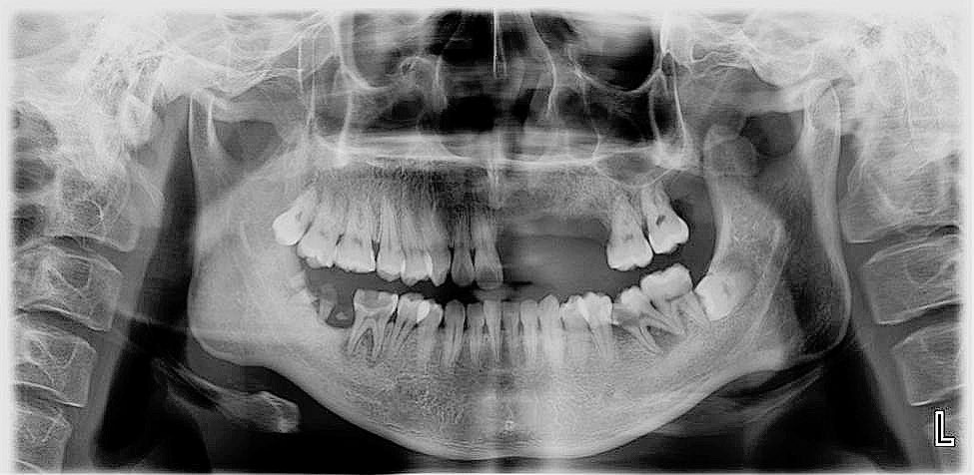
Panoramic radiographs showed the first and second upper left molars in position, accompanied by the loss of distal alveolar bone of the second upper left molar

However, the left upper lateral incisor tooth was lost, and a sequestrum was detected in the edentulous area one month after the initial tooth loss (Fig. 3A). Subsequently, complete resection of the sequestrum was performed under local anesthesia (Fig. 3B). Based on the patient’s medical history and clinical manifestations related to HZ infection, a diagnosis of odontoptosis and osteonecrosis caused by HZ was made. After four months, the second premolar was lost naturally. Meanwhile, the mobility of the canine, the first premolar and the third molar was grade III, and consequently had to be extracted. About one year after the initial diagnosis, the facial scars and pigmentation had completely subsided (Fig. 4A). Intraoral examination revealed full recovery of damaged mucosa (Fig. 4B). Panoramic radiographs demonstrated proper positioning of both upper left first and second molars; however, distal alveolar bone loss around the second upper left molar remained evident (Fig. 5).

## Discussion

The occurrence of HZ is believed to be caused by the reactivation of a previous VZV infection [12], as observed in our case. It is widely acknowledged that the first description of bone alterations associated with an episode of shingles was reported by Rose et al. in 1908 [13–15]. Subsequently, in 1922, Gonnet et al. described the initial case of alveolar osteonecrosis following zoster infection [13, 15–17], and by now, this remains recognized as rare and lesser-known [13]. The prevalence of HZ is higher among older adults, particularly individuals aged between 50 and 80 years, with an incidence rate ranging from 5 to 10 per 1,000 people [18]. In this current literature review, Table 1 summarizes 58 cases since 1955, including our case. The distribution of patients’ age groups was as follows: younger than 20 years old accounted for 5.17%, between 20 and 50 years old accounted for 32.76%, and between 50 and 80 years old accounted for 51.72%. One case (1.72%) involved a patient over 80 years old; the specific age was not mentioned in five cases (8.62%). The mean age was 48.36 years old. The acquisition of full text or even abstracts was unsuccessful in certain specific scenarios, thereby preventing the inclusion of partial specific descriptions in those articles [17, 19–30]. Among the cases of simultaneous spontaneous tooth loss and osteonecrosis following HZ infection, it was observed that only individuals without any underlying systemic diseases were affected, including two adolescent girls [16, 31] and the 29-year-old man. The youngest recorded case of tooth loss and alveolar bone necrosis caused by the VZV was identified in a 12-year-old adolescent girl [31]. There is currently no definitive evidence or laboratory markers to support a correlation between tooth loss, bone necrosis, hormone levels during adolescence, or individual immune response in this patient. The patient in this case report, a 29-year-old man with no other systemic diseases, presented with a pre-existing cough prior to the onset of HZ. Regrettably, he experienced the loss of six upper left teeth and osteonecrosis. When branches of the trigeminal nerve are involved, shingles lesions may manifest on various areas such as the face, mouth, eyes, or tongue [32]. In this literature review, HZ infection affected the maxillary branch of the trigeminal nerve in 24 cases, the mandibular branch in 29 cases, and the ophthalmic branch in only 5 cases. The specific branch affected was not mentioned in 12 cases. Typically progressing through three distinct stages: (1) prodrome, (2) active stage (or acute stage), and (3) chronic phase (also known as postherpetic neuralgia), which represents a significant complication arising from HZ infection [25, 32–35].

**Table 1:** Review of literature related to simultaneous spontaneous tooth exfoliation and osteonecrosis caused by HZ infection

Year	Author	Number of cases	Sex	Age	Bone necrosis	Tooth exfoliation /tooth position	Affected nerve	Systemic disease
1955 [32]	Dechaume et al. [19].	1	Male	48	+	+/32 33 35 36 37	No provided	Chronic hepatitis, anemia, leukopenia
1959	Delaire and Billet [17]	1/2	Female	71	+	+/45	No provided	No provided
		2/2	Female	79	+	+/41–46	No provided	No provided
1964	Delaire et al. [60].	1	Female	elder	+	+/No provided	No provided	No provided
1974	H.David Hall [53]	1	Female	62	+	+/12–18	Right trigeminal nerve ocular and maxillary divisions	Malignancy, corticosteroid therapy, and prior irradiation of maxilla
1976 [32]	Chenitz [21]	1	Male	15	+	+/22–25	No provided	Disseminated Hodgkin’s disease, stage IV-B, chemotherapy
1976	Vickery and Midda [54]	1	Female	41	+	+/31–37	Maxillary and mandibular divisions of the trigeminal nerve	Disseminated Hodgkin’s disease, stage IV-B, Combined chemotherapy
1977 [32]	Cooper, J. C [22].	1	Male	76	+	+/23 24 25	No provided	No provided
		1	male	85	+	+/41–45	No provided	No provided
1979	Delbrouck-Poot and Reginsterr [23]	1	Male	44	+	+/No provided	No provided	Hodgkin’s disease
1982	O. SCHWARTZ [25]	1	Female	66	+	+/44	Right mandibular division of the trigeminal nerve	No obvious ill
1983	Wright WE [15]	1	Female	56	+	+/21 22 23 24 25 26 27	Left trigeminal nerve ocular and maxillary divisions	Combination chemotherapy and prophylactic brain irradiation treating diffuse histiocytic lymphoma (T cell, immunoblastic)
1985	Ben-Zion Garty [31]	1	Female	12	+	+/11 12 13 14 15 16 17	Right maxillary division of the trigeminal nerve	No obvious ill
1986	H J Manz [61]	1	Female	60	+	+/two lower right teeth	Right mandibular division of the trigeminal nerve	Polymyalgia rheumatica
1987	RESA MOSTOFI [43]	1	Female	56	+	+/21 22 23 25 26 27	Left maxillary division of the trigeminal nerve.	Chronic myelogenous leukemia, mitral stenosis, left heart failure and cardiomegally.
1990	TOSHITAKA MUTO [38]	1	Male	72	+	+/41 42	Right mandibular division of the trigeminal nerve	Surgery for gastric cancer 3 years ago
1992 [32]	Peñarrocha M [27]	1	Male	50	+	+/12–18	No provided	No provided
1992	Sheldon M. Mintz [44]	1	Male	50	+	+/12	Right maxillary division of the trigeminal nerve.	No provided
1997	M L Chindia [62]	1	No provided	No provided	+	+/No provided	No provided	HIV related
1999	F J Owotade [63]	1	Male	45	+	+/two teeth	Right maxillary division of the trigeminal nerve.	No provided
2002 [18, 64]	Preeti Volvoikar [30]	1	No provided	No provided	+	+/two teeth	No provided	No ill
2003	M.A. Pogrel [65]	1	Male	51	+	+/24	Left maxillary division of the trigeminal nerve	No provided
2004	Junko Arikawa [36]	1	Male	74	+	+/43 42 41	Right mandibular division of the trigeminal nerve	Pharyngeal cancer and radiation therapy
2005	Mendieta C [32]	1	Female	63	+	+/ 43 44	Right mandibular division of the trigeminal nerve	No provided
2005	Willie F. van Heerden [39]	3	No provided	No provided	+	+/no provided	Mandibular division of the trigeminal nerve	HIV
2006	Shabnum Meer [37]	1	Male	74	+	+/31 36 35	Left mandibular division of the trigeminal nerve	Cytomegalovirus infection, diabetes
2006	P Siwamogstham [11]	2/4	Male	31	+	+/41	All three divisions of the right trigeminal nerve	HIV
		3/4	Male	29	+	+ 45	All three divisions of the right trigeminal nerve	HIV
		4/4	Female	31	+	+/32 34	Left mandibular division of the trigeminal nerve	HIV
2006	Kamala G Pillai [66]	1	Male	34	+	+/22 23 24 25	Left maxillary and ophthalmic divisions of the fifth cranial nerve	Unremarkable
2008	L Feller [67]	1	Female	30	+	+/32	Left mandibular division of the trigeminal nerve	HIV
2009	Nagaraju Kamarthi [50]	1	Male	43	+	+/17 43 44 45 46 47	Mandibular and maxillary divisions of the trigeminal nerve	HIV
2010	Veeranna Guledgud Mahima [68]	1	Male	60	+	+/44 45 46	Not provided	Unremarkable
2010	Manoj Kumar Jain [55]	1	Male	65	+	+/41–47	Right mandibular division of the trigeminal nerve	Asthma
2012	Nam-Kyoo Kim [58]	1/3	Male	78	+	+/44 45	Maxillary and mandibular divisions in the right trigeminal nerve	No provided
		2/3	Male	77	+	+/43 44 45	Rright trigeminal mandibular division	No provided
2012	Sanjog O. Chandak [69]	2/3	Male	58	+	+ /43 44 45	Right trigeminal mandibular division	No ill
2012	Lambade P [70]	1	Male	52	+	+/21 24	Left maxillary and ophthalmic divisions of the trigeminal nerve	No provided
2013	VikramK Mahajan [71]	1/3	Male	80	+	+/11–18	Right maxillary division of the trigeminal nerve	No provided
		3/3	Male	45	+	+/41–44	Right mandibular division of the trigeminal nerve	No provided
2013	Pushpanshu, K [72]	1	Female	Young	+	+/no provided	Left maxillary division of the trigeminal nerve	No provided
2014	Nicolas Cloarec [73]	1	Male	50	+	+/43 44	Right mandibular division of trigeminal nerve	HIV, Ramsay–Hunt syndrome
2014	Travis Rudd [9]	1	Male	59	+	+/41–45	Right mandibular division of the trigeminal nerve	Polyangiitis(Wegener), hypertension, and hyperlipidemia.
2015	Preeti Chawla rora [74]	1	Male	58	+	+/41–46	Right mandibular division of the trigeminal nerve	HIV
2015	Swati Gupta [64]	1/2	Female	70	+	+/16	Right maxillary division of the trigeminal nerve	No provided
2015	Santosh Patil [75]	1	Male	58	+	+/11	Right maxillary division of the trigeminal nerve	No provided
2015	Kenya Okumura [76]	1	Male	72	+	+/35	Left mandibular division of the trigeminal nerve	No provided
2016	Rupinder Kaur [77]	1	Male	47	+	+/21	Left maxillary division of the trigeminal nerve	No provided
2016	Mahdi Gholami [10]	1/2	Female	53	+	+/41 42 43	Right mandibular division of the trigeminal nerve	No provided
		2/2	Male	54	+	+/34 35	Left maxillary and mandibular divisions of the trigeminal nerve	No provided
2019	Vidya Ajila [78]	1	Female	35	+	/11–14	Right axillary division of the trigeminal nerve	HIV
2019	Yo-wei Chen [79]	1	Male	58	+	/44 45 48	Righ mandibular division of the trigeminal nerve	Erythema mutiforme
2020	Ingrid Silva Santos [80]	1	Male	57	+	+/21 31	Left maxillary and mandibular divisions of the trigeminal nerve	No provided
2020	Garima Singh [16]	1	Female	13	+	+/11	Right maxillary division of the trigeminal nerve	No provided
2021	Jian Sun	1	Male	29	+	+/21 22	Right maxillary division of the trigeminal nerve	No ill
2022	Amirthaleka Muthu Pannerselvam [81]	1	Male	25	+	+/47	Right mandibular division of the trigeminal nerve	HIV
2022	M.Chandra Sekhar [82]	1	Male	54	+	+/41–44	Right mandibular division of the trigeminal nerve	Uncontrolled diabetes for nine years
2022	Maojia Yin [83]	1	Male	50	+	+/41–44	Right mandibular division of the trigeminal nerve	No ill

The pathogenesis underlying osteonecrosis concerning HZ remains a subject of controversy, believed to be influenced by multiple factors. Some scholars suggested that osteonecrosis in HZ infection may arise due to edema resulting from inflammation-induced compression of the alveolar artery within the narrow maxillary or mandibular canal, leading to ischemia and subsequent necrosis of both periodontal ligament and alveolar bone [36, 37]. However, infection affecting the terminal nerves responsible for supplying the periosteum and periodontium within the affected dermatome area may also result in osteonecrosis [38]. It should be noted that the sympathetic nervous system plays a role in peripheral vascular bed vasoconstriction and is regulated by local vasoneural signaling mechanisms [39]. Additionally, several studies have provided genetic, neuroanatomical, and physiological evidence supporting the role of leptin in regulating bone mass through the modulation of sympathetic activity [40]. Osteonecrosis is commonly observed in patients with vascular damage resulting from aging, radiation exposure, or chronic inflammation, thereby substantiating the involvement of vascular alterations in the pathogenesis of osteonecrosis [41–43]. It’s worth noting that denervation of bone is unlikely to cause bone necrosis [44]. In this case, numerous ulcers on the lip and palate may have indicated impaired local blood flow, potentially suggesting impaired microcirculation. Impaired blood flow within the periodontal membrane results in diminished proprioception of teeth, leading to necrosis of the periodontal membrane and subsequent alveolar osteonecrosis. Meanwhile, direct viral invasion or segmental granulomatous vasculitis can contribute to the rapid progression of osteonecrosis [32]. The virus spreads through the bloodstream and lymphatic system before extending from capillaries to the epidermis where it replicates and destroys basal cells [45]. Considering the presence of cutaneous lesions as well as those on the palate, it is evident that a large number of viruses extensively affected nerves and blood vessels following a similar pathological process as described above, ultimately resulting in tooth loss and bone necrosis.

According to the literature, infection with HZ can potentially cause periodontal damage, dental malocclusion, or tooth resorption. Moreover, periodontitis or pulpitis may also contribute to jaw osteonecrosis [46–48]. Tooth loss was observed as an early indicator of postherpetic osteonecrosis [49]. The occurrence of this phenomenon was confirmed in our case. Furthermore, it is undeniable that tooth loss worsened as osteonecrosis progressed. Simultaneously, poor oral hygiene and systemic periodontitis in this patient could serve as additional contributing factors associated with tooth loss and alveolar osteonecrosis. Systemic viral infection can damage odontoblasts and induce degenerative tissue changes that ultimately lead to pulp necrosis [44, 50, 51]. However, some researchers have suggested that local dental issues might not play a significant role in most edentulous patients included in their study [52]. Meanwhile, based on a previous investigation, HZ alone is insufficient in most cases to induce these complications [53]. Vickery and Midda proposed that post-HZ damage may affect periodontal structures, making them unable to withstand mastication forces that can lead to tooth exfoliation [54]. Additionally, other studies have suggested that pre-existing pulp or periodontal inflammatory conditions or surgical procedures performed at sites of HZ infection [15, 22], or systemic viral infections affecting odontoblasts may result in degenerative tissue changes leading to more destructive alveolar osteonecrosis [55]. In this case, the affected tooth had already exfoliated before the patient’s presentation. Limited information could be obtained regarding the pulp or periodontal condition of the involved tooth. And, the patient reported developing periodontal pyorrhea after HZ infection, with severe pain leading to inadequate daily oral hygiene practices, which further exacerbated tooth loss and bone necrosis.

The prevailing consensus is that the most effective way to reduce the incidence of HZ and postherpetic neuralgia is prophylactic vaccination against HZ, with a standalone risk reduction of 70–90% [18, 56]. It is crucial to acknowledge that unclear pathogenesis, delayed diagnosis, imprecise treatment, as well as poor oral hygiene present significant barriers for specialists in managing patients experiencing spontaneous exfoliation and osteonecrosis following HZ infection. Treatment of shingles infection involves the administration of antiviral drugs, which have demonstrated efficacy in reducing both rash duration and associated pain severity, as previously mentioned. However, it is important to note that this benefit has only been observed in patients initiating antiviral therapy within 72 h from rash onset, according to the previous report [57]. Unfortunately, this patient presented to the clinic 4 days following the onset of erythema, blisters, and pain on the left side of the face. The limited dissemination of osteonecrosis in the anterior region to other regions in our reported case may be attributed to prompt sequestrum removal, effective treatment, and enhanced oral hygiene. Timely initiation of antiviral therapy and active analgesic therapy is crucial in preventing complications related to osteonecrosis following HZ. In cases where secondary infection accompanies osteonecrosis, it is necessary to perform isolation excision, removal of inflammatory tissue, and regular patient follow-up [32, 55, 58].

When rare complications such as jaw osteonecrosis with tooth exfoliation occur, aggressive use of painkillers along with adjustment of local factors and appropriate extraction of dead bone and affected teeth can lead to improved treatment outcomes. Education regarding the uncommon complications associated with trigeminal herpes zoster infection remains necessary. Trigeminal shingles fall within the diagnostic scope of all dentists and dental specialists; therefore a comprehensive understanding of this condition will help prevent unnecessary delays in treatment particularly during the prodromal stage when toothache may be the sole symptom. This poses a diagnostic challenge for clinicians who are unfamiliar with trigeminal shingles [59].

## Conclusion

In summary, we presented a rare case of maxillary osteonecrosis and tooth exfoliation induced by herpes zoster infection in a 29-year-old Chinese male without any systemic diseases. It is crucial for healthcare professionals, particularly dentists, dermatologists, and pain specialists, to be aware of the precursor symptoms and signs to avoid unnecessary actions or treatment delays. HZ infection of the face should be taken very seriously to obtain prompt treatment to prevent the rare complications of bone necrosis and tooth loss as much as possible.

## Data Availability

The datasets used and/or analyzed during the current study are available from the corresponding author on reasonable request.

## Abbreviations

HZ: Herpes Zoster
VZV: Varicella-Zoster Virus
